# Adherence and outcomes to direct oral anticoagulants among patients with atrial fibrillation: findings from the veterans health administration

**DOI:** 10.1186/s12872-017-0671-6

**Published:** 2017-09-02

**Authors:** Ryan T. Borne, Colin O’Donnell, Mintu P. Turakhia, Paul D. Varosy, Cynthia A. Jackevicius, Lucas N. Marzec, Frederick A. Masoudi, Paul L. Hess, Thomas M. Maddox, P. Michael Ho

**Affiliations:** 1 Division of Cardiology – Campus Box B130, University of Colorado Anschutz Medical Campus, 12631 E. 17th Avenue, Aurora, CO 80045 USA; 2 VA Eastern Colorado Health Care System, Denver, CO USA; 3 Veterans Affairs Palo Alto Health Care System, Palo Alto, CA USA; 4 Stanford University School of Medicine, Stanford, CA USA; 5 VA Greater Los Angeles Healthcare System, Institute for Clinical Evaluative Sciences, Western University of Health Sciences, Los Angeles, CA USA; 6 Washington University School of Medicine, St. Louis, MO USA

**Keywords:** Medication adherence, Atrial fibrillation, Direct oral anticoagulants

## Abstract

**Background:**

The direct oral anticoagulants (DOACs) reduce the risk of stroke in moderate to high-risk patients with non-valvular atrial fibrillation (AF). Yet, concerns remain regarding its routine use in real world practice. We sought to describe adherence patterns and the association between adherence and outcomes to the DOACs among outpatients with AF.

**Methods:**

We performed a retrospective cohort study of patients in the VA Healthcare System who initiated pharmacotherapy with dabigatran, rivaroxaban, or apixaban between November 2010 and January 2015 for non-valvular AF with CHA_2_DS_2_-VASc score ≥ 2. Adherence was determined using pharmacy refill data and estimated by the proportion of days covered (PDC) over the first year of therapy. Clinical outcomes, including all-cause mortality and stroke, were measured at 6 months and used to assess measures of adherence for each DOAC.

**Results:**

A total of 2882 patients were included. Most were prescribed dabigatran (72.7%), compared with rivaroxaban (19.8%) or apixaban (7.5%). The mean PDC was 0.84 ± 0.20 for dabigatran, 0.86 ± 0.18 for rivaroxaban, and 0.89 ± 0.14 for apixaban (*p* < 0.01). The proportion of non-adherent patients, PDC <0.80, was 27.6% for all and varied according DOAC. Lower adherence to dabigatran was associated with higher risk of mortality and stroke (HR 1.07; 1.03–1.12 per 0.10 decline in PDC).

**Conclusions:**

In a real-world VA population being prescribed anticoagulation for AF, more than one quarter had sub-optimal adherence. Lower adherence was associated with a higher risk of mortality and stroke. Efforts identifying non-adherent patients, and targeted adherence interventions are needed to improve outcomes.

## Background

Atrial fibrillation (AF) is the most common cardiac arrhythmia, with around 5 million new cases each year [1]. Anticoagulation significantly reduces the incidence of clinical stroke and mortality among patients with AF at moderate to high risk of thromboembolic events (CHA_2_DS_2_-VASc ≥2) [2]. Based on the results of multiple randomized controlled trials (RCTs), the direct oral anticoagulants (DOACs) are now frequently used to reduce this risk [3–5]. Additionally, they have demonstrated favorable risk-benefit profiles compared to warfarin with significant reductions in stroke, intracranial hemorrhage, and mortality [6].

While decades of knowledge have demonstrated the safety and efficacy of warfarin, less is known about how the DOACs have been adopted into routine clinical care. Furthermore, concerns remain about DOACs because of their shorter half-lives and the potential for reductions in effectiveness with poor adherence. Because of the lack of laboratory monitoring to assess therapeutic levels, it is important to assess adherence to DOACs and the extent to which it varies by patient characteristics and different DOACs currently available. The VA health care system, being the largest integrated healthcare system with an anticoagulation infrastructure and standard copayments, offers a unique opportunity to examine real-world adherence under ideal conditions.

Accordingly, we evaluated adherence patterns to the three approved DOACs (as of January 2015) and the association between adherence with outcomes among patients with AF and prescribed DOACs in the VA Healthcare System. Specifically, we sought to characterize adherence using portion of days covered (PDC) to dabigatran, rivoraxaban, and apixaban separately. Second, we assessed the patient level factors associated with nonadherence. Finally, we assessed the association between lower adherence and outcomes of stroke and mortality for each of the DOACs.

## Methods

### Study design

We performed a retrospective cohort study of patients in the VA Healthcare System who initiated pharmacotherapy with dabigatran, rivaroxaban, or apixaban between November 2010 and January 2015 for non-valvular AF with CHA_2_DS_2_-VASc score ≥ 2. Given its recent approval, edoxaban was not included in this study due to the time during which this analysis was performed. Patients were included who had the earliest DOAC prescription in November 2010 and those who were on warfarin from June, 2006. Patients with AF or atrial flutter were identified from the VA Corporate Data Warehouse medical files through a principal or secondary diagnosis of AF (ICD-9 code 427.3, 427.31, 427.32). Patients could have been taking warfarin previously and switched to a DOAC, or started on a DOAC de novo. Because we were interested in assessing adherence behavior to DOACs, we excluded patients with less than 1 year follow-up after starting a DOAC, patients who initiated a DOAC and then crossed over to warfarin, and patients who were started on 2 or more different DOACs during follow-up.

### Medication adherence

Adherence to each DOAC was calculated in the first year of therapy. Adherence was measured using the proportion of days covered (PDC), which is a validated measure previously correlated with DOAC outcomes [3, 4]. The PDC was defined as the number of doses dispensed in relation to the dispensing period. [5, 6] The numerator was based on the prescription fill dates and number of pills dispensed to determine the number of outpatient days for which each DOAC was supplied. Patients were considered adherent if they achieved a PDC > 80%, a commonly used standard [7]. As a sensitivity analysis, differences in DOAC adherence stratified by prior warfarin use was also evaluated.

### Mortality and stroke

We assessed a composite of all-cause mortality and stroke as the primary outcome of interest. Mortality was obtained through the VA Vital Status File, which compiles data from the *Beneficiary Identification Records Locator Subsystem* Death File, VA Medicare Vital Status File, and the Social Security Administration Death Master File. Stroke (ischemic and hemorrhagic) was obtained using previously validated primary or secondary *International Classification of Diseases, 9th Revision, Clinical Modification (ICD-9-CM)* diagnostic codes (346.60, 346.61, 346.62, 346.63, 431, 433.01, 433.11, 433.21, 433.31, 433.81, 433.91, 434.01, 434.11, 434.91, 997.02) [8].

### Statistical analysis

Testing for differences in the proportions of dichotomous covariates across the 3 DOAC drugs were calculated with the network algorithm of Mehta and Patel, rather than the less efficient Freeman-Halton extension to 2 × 3 tables of the Fisher exact test. The one-way analysis of variance Savage score was used to test for differences in location for the continuous covariates. A Generalized Estimating Equation (GEE) logistic regression with adjustment for risk factors and a term to adjust for patients clustered within hospitals was used to evaluate the interaction of prior warfarin use vs. de novo treatment with DOAC type and to determine significant risk factors for the outcome of nonadherence. Similarly, a GEE linear model was used to determine the interaction effect of prior warfarin use on PDC at one year.

Cox proportional hazards models adjusted for patient risk factors (heart failure, age, diabetes, stroke/transient ischemic attack, vascular disease, sex, coronary artery disease), and a term for patients within hospital (frailty, a standard statistical term in time to event analyses indicating adjustment for correlated random effects), were used to assess measures of PDC at 6 months and nonadherence at 6 months on the combined endpoint of mortality and/or stroke for each DOAC. The results for mortality and stroke for apixaban were not included due to the low event rate.

All analyses were performed using SAS9.4 TS Level 1 M3 software, © 2002–2012 by SAS Institute Inc., Cary, NC, USA. The Colorado Multiple Institutional Review board approved this study and waiver of informed consent was granted. The authors are solely responsible for the design and conduct, drafting, and editing of this manuscript and its contents.

## Results

**Fig. 1:**
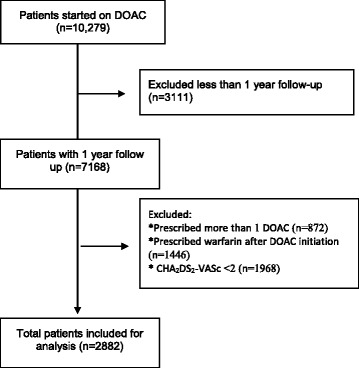
Flow Diagram of patient inclusion and exclusion

Over the study period, a total of 10,279 patients were started on DOACs. After excluding patients with less than 1-year follow-up period (*n* = 3111), those who were prescribed more than 1 DOAC during the study period (*n* = 872), those who were prescribed warfarin after DOAC initiation (*n* = 1446) and those with CHA_2_DS_2_-VASc score < 2 (*n* = 1968), a total of 2882 patients were included in the analysis (Fig. 1).

Baseline patient characteristics are described in Table 1. The mean age of the cohort was 67.4 ± 9.5, most whom were white (82.7%) and male (96.9%). Co-morbidities included hypertension (88.7%), diabetes mellitus (48.5%), congestive heart failure (29.6%), cerebrovascular disease (11.1%), and prior myocardial infarction (13.6%). The mean and median CHA_2_DS_2_-VASc score were 2.9 ± 1.1 and 3 (IQR 2–3), respectively. Most patients in the cohort were prescribed dabigatran (72.7%), compared with rivaroxaban (19.8%) or apixaban (7.5%). Differences in patient level characteristics between DOACs were largely similar with significant differences seen in age (mean age among patients prescribed dabigatran 66.9 vs. 73.1 for apixaban), rate of hypertension (89.4% for dabigatran vs. 83.3% for apixaban), and CHA_2_DS_2_-VASc score (2.89 for dabigatran vs. 3.2 for apixaban).

**Table 1:** Baseline characteristics among patients being prescribed DOACs

Patient Characteristics	All *n* = 2882	Dabigatran *n* = 2096 (72.7%)	Rivaroxaban *n* = 571 (19.8%)	Apixaban *n* = 215 (7.5%)	*p*
Age (mean + SD)	67.4 (9.5)	66.9 (9.3)	67.3 (9.7)	73.1 (8.8)	<0.01
Male (%)	2792 (96.9)	2035 (97.1)	552 (96.7)	205 (95.4)	0.32
White Race (%)	2383 (82.7)	1735 (82.8)	467 (81.8)	181 (84.2)	0.72
Hypertension (%)	2556 (88.7)	1874 (89.4)	503 (88.1)	179 (83.3)	0.03
Congestive Heart Failure (%)	852 (29.6)	624 (29.8)	173 (30.3)	55 (25.6)	0.41
Diabetes Mellitus (%)	1398 (48.5)	1036 (49.4)	270 (47.3)	92 (42.8)	0.15
Cerebrovascular Accident (%)	321 (11.1)	240 (11.5)	63 (11.0)	18 (8.4)	0.41
Prior Myocardial Infarction (%)	391 (13.6)	269 (12.8)	89 (15.6)	33 (15.4)	0.16
Peripheral Arterial Disease (%)	280 (9.7)	201 (9.6)	53 (9.3)	26 (12.1)	0.45
Peripheral Arterial Disease (%)	2.9 (1.1)	2.9 (1.0)	3.0 (1.1)	3.2 (1.2)	<0.01
CHA_2_DS_2_-VASc (mean + SD)	2.9 (1.1)	2.9 (1.0)	3.0 (1.1)	3.2 (1.2)	<0.01
Adherence					
Mean (SD) pill count per dispensed supply	38.1 (20.9)	38.2 (20.8)	38.2 (22.0)	36.4 (18.7)	<0.01
Proportion of Days Covered (mean + SD)	0.85 (0.19)	0.84 (0.20)	0.86 (0.18)	0.89 (0.14)	<0.01
PDC < 80% n (%)	796 (27.6%)	604 (28.8%)	143 (25.0%)	49 (22.8%)	0.05

The mean PDC was 0.84 ± 0.20 for dabigatran, 0.86 ± 0.18 for rivaroxaban, and 0.89 ± 0.14 for apixaban (*p* < 0.01) (Table 1). The proportion of non-adherent patients, defined as PDC <0.80, was 28.8%, 25.0%, 22.8% respectively (*p* = 0.05). Adherence using PDC stratified by patients who had previously been on warfarin and those who were started de novo are described in Table 2. There was a statistically significant small difference in PDC between drug types and history of prior warfarin use (*P* < 0.01). Based on PDC <80% for nonadherence, there was no statistically significant difference between drug types and history of prior warfarin use (*P* = 0.44).

**Table 2:** Adherence patterns among patients previously on warfarin and those started de novo

	Dabigatran	Rivaroxaban	Apixaban	p
De novo (Mean, SD)	0.84 (0.20)	0.87 (0.19)	0.87 (0.15)	<0.01
Prior warfarin use (Mean, SD)	0.84 (0.19)	0.86 (0.18)	0.89 (0.14)	<0.01

Table 3 describe factors associated with nonadherence, where an odds ratio of >1 is associated with greater nonadherence and an odds ratio < 1 is associated with greater adherence. Age in years (OR, 0.98; 95% CI, 0.96–0.99; *p* < 0.01), hypertension (OR, 0.69; 95% CI, 0.49–0.99; *p* = 0.04), diabetes (OR, 0.57; 95% CI, 0.41–0.79; *p* < 0.01), and stroke (OR, 0.36; 95% CI, 0.2–0.68; *p* < 0.01) were associated with greater adherence.

**Table 3:** Predictors of medication nonadherence

	All Drugs (Dabigatran reference drug)	Dabigatran	Rivaroxaban	Apixaban
Parameter	Odds Ratio (95% CI)	Odds Ratio (95% CI)	Odds Ratio (95% CI)	Odds Ratio (95% CI)
Age	0.98 (0.96, 0.99)	0.99 (0.97, 1.00)	0.96 (0.93, 0.99)	0.97 (0.90, 1.04)
Male	0.85 (0.54, 1.34)	0.85 (0.47, 1.53)	1.58 (0.54, 4.61)	0.26 (0.06, 1.13)
White Race	0.73 (0.59, 0.92)	0.69 (0.53, 0.90)	0.81 (0.48, 1.37)	0.61 (0.23, 1.65)
Hypertension	0.69 (0.49, 0.99)	0.74 (0.47, 1.16)	0.64 (0.27, 1.50)	0.66 (0.17, 2.61)
Congestive Heart Failure	0.74 (0.53, 1.03)	0.91 (0.61, 1.36)	0.52 (0.24, 1.13)	0.28 (0.07, 1.10)
Diabetes Mellitus	0.57 (0.41, 0.79)	0.68 (0.47, 0.97)	0.56 (0.27, 1.14)	0.15 (0.03, 0.67)
CVA	0.36 (0.20, 0.68)	0.47 (0.23, 0.94)	0.29 (0.06, 1.40)	0.11 (0.01, 1.43)
Prior Myocardial Infarction	1.20 (0.96, 1.50)	1.40 (1.07, 1.82)	0.88 (0.55, 1.39)	0.53 (0.18, 1.53)
Peripheral Arterial Disease	0.99 (0.72, 1.36)	0.96 (0.68, 1.34)	1.14 (0.60, 2.17)	1.32 (0.49, 3.54)
CHA_2_DS_2_-VASc Score	0.88 (0.76, 1.01)	0.81 (0.68, 0.97)	1.09 (0.85, 1.41)	0.81 (0.40, 1.64)

The combined end-point of mortality and/or stroke based on adherence patterns are described in Table 4. The total number of follow-up days was 1,922,857, with mean (SD) and median (IQR) of 667.2 (432.2) and 582 (388, 933), respectively. The overall rate of death and stroke were 17.4% (502) and 1.7% (49), respectively.

**Table 4:** Time fixed analysis for mortality and stroke based on adherence

	Dabigatran		Rivaroxaban	
	HR (95% CI)	p	HR (95% CI)	p
PDC 6 month (per 0.1 decline)	1.07 (1.03–1.12)	<0.01	1.07 (0.89–1.28)	0.46
Nonadherence 6 month	1.54 (1.20–1.97)	<0.01	1.74 (0.77–3.94)	0.18

Models were adjusted for demographics and comorbidities (heart failure, age, diabetes, stroke/transient ischemic attack, vascular disease, sex, coronary artery disease)

Among patients on dabigatran, there was a significant association between lower adherence (per 0.1 decline in PDC measured over the initial 6 months after initiation of therapy) and higher risk of death or stroke; (HR, 1.07; 95% CI, 1.03–1.12; per 0.1 drop in the PDC; *p* < 0.01; *n* = 277 total events; death = 253, stroke = 24). Among patients on rivaroxaban, there was a trend for an association between lower adherence at 6 months (per 0.1 decline in PDC) and higher risk of mortality and stroke (HR, 1.07; 95% CI, 0.89–1.28; *p* = 0.46; *n* = 25 total events; death = 24, stroke = 1]. In secondary analysis, nonadherence (PDC <80%) at 6 months to dabigatran was associated with increased risk of death or stroke (HR, 1.54; 95% CI, 1.20–1.97; *p* < 0.01). There was a similar trend for rivaroxaban but it was not statistically significant (HR, 1.74; 95% CI, 0.77–3.94; *p* = 0.18). Outcomes analyses were not conducted for apixaban due to the small number of events (*n* = 5).

## Discussion

Using VHA data from June 2006 to January 2015, we characterized adherence to DOACs and assessed the association between nonadherence and outcomes among patients in the VA Healthcare System. There were three main findings. First, 1 in 4 patients had sub-optimal adherence, which varied slightly based on the DOAC. Second, several patient factors were associated with greater medication adherence, including older age, diabetes, and stroke. Third, nonadherence was associated with adverse outcomes including mortality and stroke for dabigatran, with a similar trend for rivoraxaban.

Medication nonadherence is a prevalent and growing concern among healthcare providers. Previous investigations have described adherence patterns to dabigatran. Using the Danish National Prescription Registry, Gorst-Rasmussen evaluated 2960 patients started on dabigatran for AF [9]. The mean one-year PDC was 0.84 and about one-quarter of patients were nonadherent (PDC <80%). Among 17,000 patients on dabigatran, PDC among de novo starters were 0.67 and 0.71 for those previously on warfarin [10]. Schulmann interviewed a small cohort of patients (103) and found that 88% of patients were adherent (PDC >80%) to dabigatran [11]. The VA Healthcare System offers a unique opportunity to examine real-world adherence under ideal conditions. It is the largest integrated healthcare system and has specific processes of care by which anticoagulation is safely administered (including anticoagulation clinics and a clinical pharmacist infrastructure). Additionally, copayments are standard across oral anticoagulation agents which does not lead to a differential copayment burden. A previous investigation in the VA population demonstrated that 27.8% of patients on dabigatran for AF were nonadherent, which was also associated with an increased risk for all-cause mortality and stroke [8]. Our findings further this knowledge by demonstrating that nonadherence is common, even among the newer DOACs. Furthermore, these findings are in direct discordance with adherence patterns seen in randomized controlled trials which are generally higher. Differences in adherence patterns between clinical trials and routine clinical practice are multifactorial and include a potential Hawthorne effect for those enrolled in clinical trials and lack of close follow up, high incidence of comorbidities and polypharmacy, and/or financial constraints for those in routine clinical care. Additional research is needed to determine which factors can be targeted to maximize patient outcomes.

Predictors of greater adherence included older age and a history of hypertension, diabetes, and stroke. Factors previously known to be associated with nonadherence include male gender, homelessness, and psychiatric disorders, particularly depression while those associated with greater adherence include high level of education, stability of family background, and affordability to therapy [12–15]. Additionally, treatment of asymptomatic disease is associated with poor adherence [15]. Importantly, cardiovascular disease, such as in some cases of atrial fibrillation, are generally asymptomatic chronic diseases where the perceived benefits of daily medical therapy may not be apparent to patients. Among these patients, physicians should have a heightened awareness of the possibility of poor adherence and consider directly asking patients about their adherence.

Adherence to DOACs was a significant predictor of outcomes including all-cause mortality and stroke. A similar association of risk of death or stroke was seen among the 27.8% of non-adherent patients on dabigatran, with a hazard ratio of 1.13 (95% CI 1.07–1.19) [8]. Furthermore, prior studies have shown similar associations of risk and nonadherence among patients with coronary artery disease, heart failure, hypertension, and hyperlipidemia [16–19]. For example, among 10,000 patients with diabetes, 21% were non-adherent which was associated with an increased risk for all-cause hospitalization and mortality [16]. These findings suggest that medication nonadherence is common among many chronic illnesses and highly impacts outcomes.

There are a few implications of these findings. First, while adherence was significantly different stratified by DOAC, the absolute difference was very small. Similarly, differences in demographic and co-morbidities associated with nonadherence were small and determining nonadherence to DOACs based on these clinical characteristics may be difficult. This suggests that other mechanisms to detect patients at risk for nonadherence need to be identified, including directly asking patients about adherence or utilizing data systems in which prescription refills, or lack thereof, are recorded and provided in real-time. Further evaluation of such data systems should be developed to determine if they can successfully be implemented and improve patient adherence. Additionally, it is often thought that adherence to medical therapy improves with simplification; however, we found that adherence to the once daily rivaroxaban did not have better adherence rate. Further studies are needed to evaluate this perception.

Certain factors should be considered in the interpretation of this study. First, our analysis was confined to patients in the VA Healthcare System with the clear majority of patients being white males. Thus, our results may not apply to the broader population of patients being prescribed DOACs. However, the VA provides an opportunity to examine adherence in a closed pharmacy system with highly reliable data sources because refill data for these medications are well captured given the smaller copay in the VA. Second, while we utilized pharmacy databases to capture medication dispensing, there is a lack of distinction between dispensing and consumption, making this type of analysis less reliable at the individual level. However, refill adherence has previously been shown to be an accurate marker of patients’ adherence in the VA system [20]. Additionally, direct methods of assessing adherence (i.e. directly observed therapy) have limitations and are not practical for routine clinical use. Third, there were relatively fewer patients on rivaroxaban and apixaban as these are newer to the VA system and their use is dependent on contraindications or previous intolerance to dabigatran. Therefore, broader examination of outcomes is limited due to lack of power given the relatively smaller sample size. Furthermore, longer term outcomes are limited given the exclusion of a large majority of patients who had less than one year follow up. Additionally, the impact of longer duration of follow up on adherence or outcomes was not addressed but should be explored in future analysis. Fourth, direct comparisons between classes of DOACs were not performed as we are unable to account for unmeasured confounders and selection bias for the determination of which DOAC was selected. Fifth, we did not evaluate the association between adherence and bleeding given the low rate of bleeding events. Sixth, a large portion of patients (1446) were excluded after having been prescribed warfarin after DOAC initiation. The reasons for such were unclear from this study and further evaluation is needed to address reasons for a change in anticoagulation strategies. Finally, our results are likely an overestimation of adherence based on the exclusions. For example, by excluding patients who stopped or did not tolerate a DOAC, we selected a group of patients more likely to adhere to medical therapy and thus increasing the adherence rate beyond what is seen in practice.

## Conclusions

This study characterizes adherence patterns and outcomes among patients in the VA Healthcare System using DOACs for AF. First, more than one quarter of patients had sub-optimal adherence with DOACs. While there were differences in adherence between DOAC and patient characteristics, these were not clinically significant. Second, outcomes including all-cause mortality and stroke were associated with medication adherence. Efforts towards identifying non-adherent patients and targeting adherence interventions are needed.

## Abbreviations

AF: Atrial fibrillation
DOAC: Direct oral anticoagulant
GEE: Generalized estimating equation
HR: Hazard ratio
IQR: Interquartile range
PDC: Proportion of days covered
RCT: Randomized controlled trial
SD: Standard deviation
